# Ethical perspectives on modifying animals: beyond welfare arguments

**DOI:** 10.1093/af/vfz055

**Published:** 2020-01-10

**Authors:** Bernice Bovenkerk

**Affiliations:** Philosophy Group, Wageningen University, Wageningen, The Netherlands

**Keywords:** biotechnology, genetic modification, good life

**Implications**

- The arguments “beyond welfare” appear to be part of broader conceptions of the “good life” and of how to be a good person.
- There is less agreement on the arguments beyond welfare, which rely on people’s comprehensive notions of the good life, about which people disagree fundamentally.
- By only taking rule-ethical principles seriously, many important values and meanings that people attach to life and to the world around them are disregarded.
- We do not blindly employ our technologies on animals, but we should once in a while step back and reflect on what modifications mean for our relationship to animals and nature and on what kind of world we want to live in.

## Introduction

**Figure 1.**
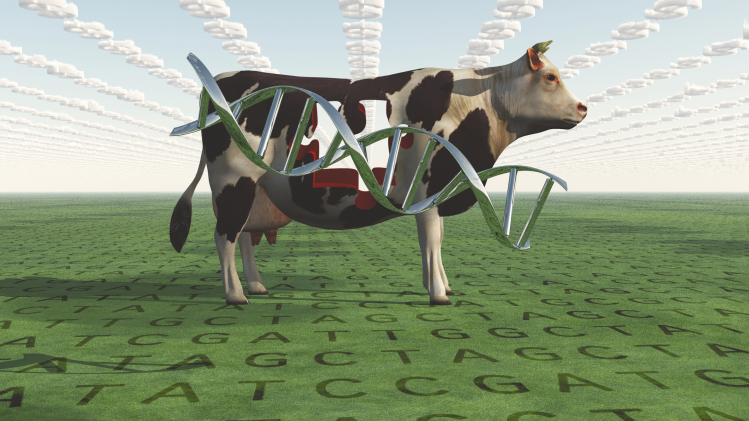
Schematic image describing genetic modification of cattle.

One of the earliest applications of biotechnology to livestock was the so-called Beltsville pigs. Researchers at the U.S. Agricultural Research Service in Beltsville, Maryland, inserted the gene for human growth hormone into pigs in order to achieve a better food conversion rate (Thompson, 1997). This led to many health and welfare problems in the pigs, such as arthritis and lung problems and it ultimately led the researchers to terminate the experiment. This was seized upon by critics of biotechnology to show that genetically modifying animals was unacceptable (Thompson, 1997). However, I don’t think the critics had a very strong case. You could say that these pigs did not present us with a moral dilemma. After all, when it is clear that something is morally wrong, it is not a moral dilemma, it is simply wrong. It was recognized by the researchers that the animals’ welfare was harmed and therefore they terminated the experiment. But in reality, most modified animals do not have welfare problems. Some modifications in fact “solve” welfare problems. Think, for example, of polled cattle (Figure 1). Because they are modified to not grow horns, they are less likely to harm other cattle and farmers. But is this then the end of the story? I don’t think so. Many people still have moral problems with modified animals, whether or not they experience welfare problems. Perhaps it would have been better for the critics to focus on those other problems.

Of course, one could argue that welfare is commonly understood in quite a narrow sense. Commonly used criteria such as The Farm Animal Welfare Council’s five freedoms (Brambel Report, 1965) do not constitute welfare, but only the “necessary conditions for welfare” (Harfeld et al., 2016). Welfare is more than what you can objectively measure. There is also a broader sense of the term welfare, which perhaps should be termed well-being. In this broader sense, welfare is not just measured at specific points in time, but over the course of the animal’s whole life. The central question then becomes “what constitutes a good life for animals”? Of course, the absence of pain and injury, hunger and thirst, fear and stress are very important, but well-being is also about things such as enjoyment, about achieving what one wants to achieve, about having good relationships with conspecifics. I don’t think all of these are necessarily covered by the five freedoms, not even by the freedom to express normal or species-specific behavior.

But still, genetic modification does not necessarily interfere with well-being in this broader sense either and the moral objections remain. Welfare or well-being are in the end about the subjective experiences of animals, but many of the moral discussions about genetically modified animals are not about how the “animals” experience it, but how we “humans” experience it. What does genetic modification do to our own view of the good life or our worldview? In other words, there are objections to genetic modification that move beyond welfare and these are the focus of this contribution. I will briefly discuss four clusters of these arguments or objections beyond welfare: the arguments that modifying animals violates their integrity, that it instrumentalizes animals, that it amounts to playing God, and that it is unnatural. These objections are actually not limited to genetic modification of animals, but often apply to modifications through artificial selection as well. For example, in my research about people’s perceptions about pedigree dog breeding, I encountered all of these objections as well. I will not present a comprehensive overview, but I do address some of the most common arguments in the area of animal ethics. Of course, there are also arguments that go beyond the field of animal ethics—such as arguments about justice between people. For example, we can wonder whether it is fair if only large livestock production facilities have sufficient means to employ certain technologies. However, in this short contribution, I have to necessarily limit myself to only animal ethical discussions.

After this overview, I will raise the question of what to make of these arguments. Most people agree that it is wrong to modify animals in such a way that it harms their welfare. But there is far less consensus about the value of these arguments beyond welfare. They can be traced to worldviews and views of the good life and this is something that people disagree about. I will argue that it is still important to discuss these publicly and not to relegate them to the private sphere.

## Integrity

One argument that has been used against modifications of animals is that they violate animals’ integrity. Even though the concept integrity has been applied in the debate about biotechnology, it was originally used to articulate more general objections to interventions that cannot be expressed in terms of harm to animal health and welfare.

Rutgers and Heeger describe integrity as “the wholeness and intactness of the animal and its species-specific balance, as well as the capacity to sustain itself in an environment suitable to the species” (1999). So, for example, a dog whose tail has been docked is violated because it is no longer intact and a Belgian Blue cow that can no longer give birth naturally cannot sustain itself in an environment suitable to its species and therefore could be said to be violated in its integrity.

At first sight, integrity may seem to refer to a biological norm. Yet, we would not speak of the violation of integrity in all cases where an animal’s intactness is violated. If we dock a dog’s tail for medical reasons, we would not speak of an integrity violation, but if we do so for aesthetic reasons, we would. This means that this notion of integrity primarily refers to the “intention” behind the interference. Thereby it is a moral rather than a biological norm. Also, integrity refers to a “species-typical norm” (Thompson, 2008). In other words, it refers to “the cowness of a cow” or “the chickenness of a chicken”. The point of reference then is not the animal itself as adapted to the farm or the home, but rather the species as it would appear in nature. Integrity, in other words, is about an ideal image that we have of how animals ought to be. This raises the question of why we would take this image of the animal as it would appear in nature as the ideal baseline. This seems to come down to the idea that animals are somehow better or more valuable, the more natural they are. Within ethical theory, it has proven difficult to give a conclusive justification for the appeal to integrity. But still, it does seem to appeal to an intuition that many people have that we should not “tamper” with animal’s genomes. Of course, appealing to an intuition in itself does not make an argument justified. However, a persistent intuition does give us reason to look for further justifications of the argument. This is especially the case with the notion of integrity, which has proven useful in practice. For example, integrity violation was one of the criteria of the Dutch Committee for Animal Biotechnology in its decision whether or not to grant a license for genetically modifying animals (Brom et al., 1997) (see also Bovenkerk, 2012).

## Instrumentalization

The idea that either genetic modification or modifications through artificial selection tends to instrumentalize animals in fact refers to a cluster of objections. Besides instrumentalization, similar terms that have been used, and that each have a slightly different theoretical basis, are objectification, commodification, alienation, and de-animalization (Bos et al., 2018). Objectification draws on the feminist literature about the objectification of women, commodification and alienation draw on the Marxist tradition, and de-animalization is a relatively new term that draws on the virtue ethical tradition and that applies particularly to the context of intensive livestock farming.

Two general meanings of instrumentalization can be distinguished: it can either mean treating an animal as an object or actually turning an animal partly into an object (Brom, 1997). Treating as if an animal is an object is problematic because it leads to a denial of its own interests or its nature. It could lead people dealing with the animal to forget about the animal’s needs and desires, and it could lead them to regard the animal as fungible; as if a particular individual can be easily replaced by another.

Turning into an object (whether intentionally or not) means that the animal is treated solely as an instrument for our use or that it is in fact turned (partly) into an artifact. This sense of the term is more common in the context of modifications, because by changing the genetic make-up of the animal, it arguably turns into an artifact, namely into something that is manufactured by humans. Modified animals become “living parts of machinery” Harfeld et al., 2016). They are adapted to such an extent that they can fit better within our production systems. So, for example, we could use CRISPR-Cas9 to breed pigs that are immune to the viral infections that often plague them at the farm. On the one hand, this would save many pigs’ lives, but on the other hand, it could be argued that there would not be so many viral infections in the first place if pigs were housed in less intensive systems. The polled cattle referred to earlier would be another example: Would it be necessary to dehorn them if they were held in a different way? Of course, the merits of these examples could be discussed empirically, but what matters here is that interventions of this kind raise the question whether we should adapt the animal to the farm or rather the farm to the animal. It could be argued that what happens in practice is in fact both: the animal is adapted to the farm and vice versa. However, the view behind the objection to instrumentalization is that the farm should always be adapted to the animal rather than the other way around. Why is instrumentalization deemed morally problematic? Because animals’ own subjectivity or autonomy is denied and they are not seen as individual beings with their own goals in life, but as tools to reach the goals of human beings. So, in the end, this objection is about the question what moral status we attach to animals and that is something about which people tend to disagree (Bovenkerk and Meijboom, 2012).

**Figure 2.**
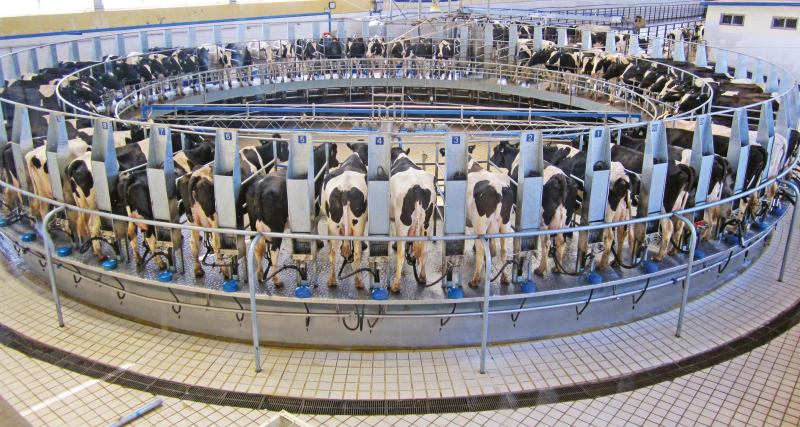
Robotic milking system for dairy cattle.

The related notion of de-animalization, which was coined by Harfeld and others, indicates that in intensive livestock farming production animals are taken out of their own evolutionary and environmental context (Figure 2) and people involved in the animal production system reduce them to a “production unit” or an artifact (Harfeld et al., 2016). They are in effect reduced to their functions within the system where there is room for exercising few other capabilities and behaviors than “giving offspring, producing milk, and dying”. This notion is in fact based on a virtue ethical argument. In short, virtue ethics argues that people should cultivate the right moral character by behaving virtuously. Harfeld et al. (2016) claim that experience of animals and their complex behavior as evolved in their natural environment is necessary for people to develop the practical wisdom that will help them build moral character and make sound judgments regarding the treatment of animals. They argue that current conditions in livestock production are detrimental to our grasping the “animalness” of animals and this makes us fail to give animals ethical consideration.

## Playing God

The argument that modifying animals amounts to playing God rejects intervention in the order of the creation. The objection to playing God expresses an intuition that certain boundaries should not be crossed by humans. The power to create lies in the hands of God and this creation should be treated respectfully by human beings. This is one possible interpretation of the argument, but this objection is usually not meant as a religious argument. In fact, in the eyes of some theologians, humans were created by God as co-creators and these theologians therefore have no problem with genetic engineering as such (van den Belt, 2009). As Brom argues, this is usually meant as a secular argument about the proper role of human beings within nature or vis-a-vis technology (Brom, 1997). By invoking this argument, critics reject the human pretension of control and almightiness that appears to lie behind certain technologies. This view was already central in the ancient Greek idea of human “hubris”—or arrogance—and is also the theme of Mary Shelley’s Frankenstein. This objection warns against the human tendency to think that nature or life can be completely manufactured or planned, and it urges us to acknowledge life’s unpredictability. This is not a knock-down argument against genetic modification as such, or against all types of modifications, but it could be seen as a warning to not expect too much control over nature. As it calls for people to practice an attitude of modesty and temperance, it could be interpreted as a virtue ethical argument.

## Unnaturalness

**Figure 3.**
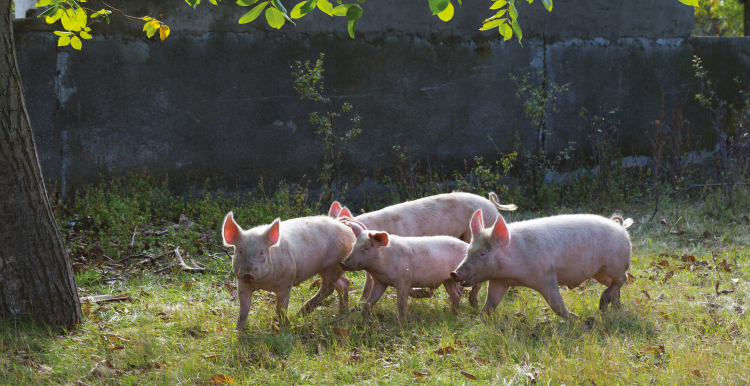
Feral domestic pigs are potential reservoirs of African swine fever virus and other zoonotic diseases.

When the argument that genetic engineering is unnatural is invoked, this often refers to the idea that certain natural boundaries have been crossed. For example, the boundaries between species. In response, it has been put forward that on a genome level, these boundaries do not really exist. But this response misses the point of the objection. The point here is not that something is done that would never happen in nature, but rather that interfering itself is deemed unnatural, because it is carried out by humans. The reference point for naturalness then seems to be the “untouched” animal, as it would appear in nature, as the end result of the process of evolution (Figure 3). The natural is then seen as opposed to either the artificial or the cultural. By invoking the unnaturalness-objection in this context, critics mean that by adapting animals, we are doing something which is artificial and/or we are turning the animal into an artifact. The argument is therefore related to the instrumentalization objection as discussed earlier.

Philosophers are often struggling with this argument. This is because appeals to nature can easily be rejected as a so-called naturalistic fallacy. If we argue from an observation about nature directly to a normative conclusion, we are said to commit the naturalistic fallacy. We should not take nature as a guide to our moral actions. Many cruel things happen in nature that we would find unethical. Some animals eat the babies of their competitors. Nobody would argue that we should do that too because it is natural. Invoking nature can also be misused for social or political goals. Think of statements such as “women should stay at home and look after the children, because it is in their nature to care” (Soper, 1995). It has often been argued that when people claim that something is unnatural, they are actually saying they find it undesirable (Zwart, 1997). In other words, rather than finding adaptations bad because they are unnatural, people call them unnatural because they think they are bad. But many unnatural things are generally considered good: wearing glasses goes against nature in a sense, but is it thereby morally problematic?

The unnaturalness objection is therefore a difficult argument. Nevertheless, in discussions about genetically modifying animals or artificial selection this argument keeps resurfacing. This argument was also encountered in my own research about breeding pedigree dogs (Bovenkerk and Nijland, 2017). Apparently, it expresses a deeply felt intuition. What could be behind this intuition? What became clear from my research is that the step from unnatural to morally wrong is often not made directly, but actually relies on underlying views on nature and our relation to animals. Several of my respondents reasoned from an attitude of respect for nature and they warned for the harmful consequences of meddling with processes we do not completely understand. They also rejected an instrumental vision of nature and animals, where animals are simply regarded as resources or tools for our purposes. Many respondents showed respect for evolutionary processes. This does not mean that they held that whatever nature produced through evolutionary processes was necessarily good or benign, but that since natural processes have been tried and tested for much longer than artificial adaptations, humans should take a more modest attitude and learn from nature rather than trying to change it. The unnaturalness-argument, then, should be understood as a way to express the meaning people attach to nature and the view they have of our role within nature, and not as a hard and fast criterion to demarcate acceptable from unacceptable actions.

## What is the Upshot of these Arguments?

**Figure 4.**
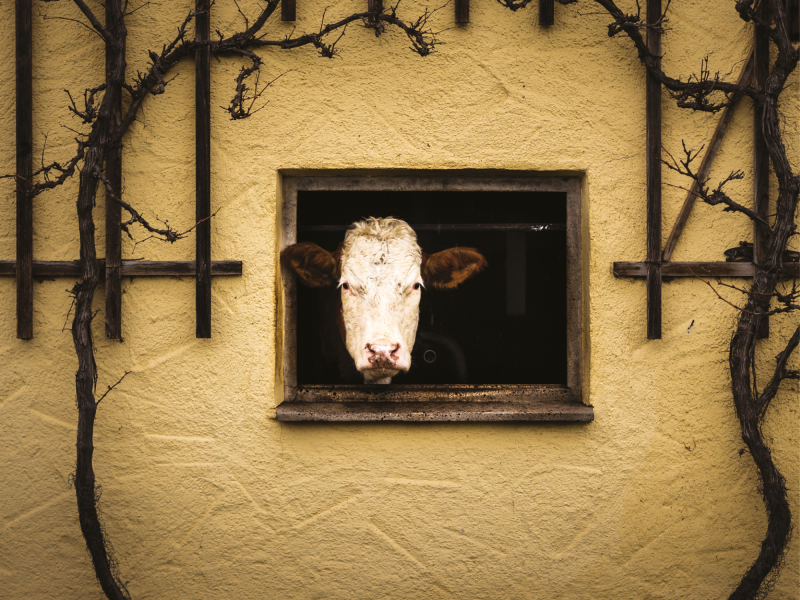
A cow looking out of a barn window.

If we look at all these arguments beyond welfare, what becomes clear is that they are based on how we view animals, how we view the human–animal–nature relationship, and more broadly on what we see as a good life. The arguments about integrity and instrumentalization are based on the view that animals have an intrinsic value, apart from their value as instruments for our use. They are also based on views about what an ideal animal is; one that is as close to its original species-specific nature as possible. If we look at arguments such as playing God and unnaturalness, they express a view about the role of humans in the natural order that should be more modest. Many of these arguments also appear to rely on virtue ethical theory, which cautions us to be temperate and find the right balance between vice and sainthood. For example, a virtuous person is brave, which means finding the right mean between cowardice and overconfidence (Figure 4).

The arguments “beyond welfare” then appear to be part of broader conceptions of the “good life” and of how to be a good person. Philosophers in this context often refer to “comprehensive notions of the good life”. These connect less to so-called “rule-ethical” theories in ethics than to so-called “life-ethical theories”. Rule-ethical theories aim to formulate impartial rules that enable peaceful cohabitation between individuals. They formulate rules that are often based on commonly held moral concepts, such as justice or freedom of choice. On the other hand, in life-ethical theories, discussions about the good life are central. Life-ethical theories ask questions such as “how do we envisage the good life for humans and animals?” and “how do we show a respectful attitude towards animals and nature?” These are not questions that people tend to agree about. Most people now agree that animal welfare is important to protect, but there is much less consensus about notions, such as integrity or naturalness.

Summing up, there is less agreement on these arguments beyond welfare, which rely on people’s comprehensive notions of the good life, about which people disagree fundamentally. They do not provide clear rules for right or wrong behavior. For this reason, they tend to be relegated to the private sphere. The idea behind this is that everyone is entitled to their private opinions about comprehensive notions of the good life, but that public decisions should not be based on them. These should only be based on views about which some form of consensus has been reached. In my view, this is problematic. By only taking rule-ethical principles seriously, many important values and meanings that people attach to life and to the world around them are disregarded. If we can only base the decision of whether or not to modify an animal on what it does to an animal’s welfare, a lot of important values are excluded. This is not to say that arguments beyond welfare lead to rules or regulations in any straightforward sense. Still, excluding them from the decision-making process altogether is also problematic as it skews the decision-making process in favor of those who are eager to implement new technologies and excludes the views of those who are more cautious (Swiertra, 2003).

In my view, these objections or arguments beyond welfare should be the subject of public debate. We should not only talk about what modifications of animals we want to forbid or not forbid, but also about the broader implications of such modifications.

In a public debate, people can explain their worldview to others. Even though people may have fundamental disagreements on the good life, it is still something that you can discuss and give arguments for (Bovenkerk, 2012). And even if they don’t come to agree, they can at least take other people’s views more seriously. This means minimally that those who modify animals should proceed with more caution. It also means that we do not blindly employ our technologies on animals, but we should once in a while step back and reflect on what modifications mean for our relationship to animals and nature and on what kind of world we want to live in.

## About the Author

**Bernice Bovenkerk** is associate professor of philosophy at Wageningen University and Research, the Netherlands. Her research and teaching deals with issues in animal and environmental ethics, the ethics of climate change, and political philosophy. Current topics are the moral status of animals and other natural entities, with a particular focus on fish and insects, the ethics of animal domestication, animal (dis)enhancement, and deliberative democracy. She received her PhD from the University of Melbourne, Australia. She received her master’s title at the University of Amsterdam. Her homepage is: bernicebovenkerk.com.
